# Heart Rate Variability in Porcine Progressive Peritonitis-Induced Sepsis

**DOI:** 10.3389/fphys.2015.00412

**Published:** 2016-01-06

**Authors:** Dagmar Jarkovska, Lenka Valesova, Jiri Chvojka, Jan Benes, Jitka Sviglerova, Blanka Florova, Lukas Nalos, Martin Matejovic, Milan Stengl

**Affiliations:** ^1^Faculty of Medicine in Pilsen, Biomedical Center, Charles University in PraguePilsen, Czech Republic; ^2^Department of Physiology, Faculty of Medicine in Pilsen, Charles University in PraguePilsen, Czech Republic; ^3^First Medical Department, Faculty of Medicine in Pilsen, Charles University in PraguePilsen, Czech Republic; ^4^Department of Anesthesia and Intensive Care Medicine, Faculty of Medicine in Pilsen, Charles University in PraguePilsen, Czech Republic

**Keywords:** sepsis, septic shock, heart rate variability, experimental model, pig, electrocardiography

## Abstract

Accumulating evidence suggests that heart rate variability (HRV) alterations could serve as an indicator of sepsis progression and outcome, however, the relationships of HRV and major pathophysiological processes of sepsis remain unclear. Therefore, in this experimental study HRV was investigated in a clinically relevant long-term porcine model of severe sepsis/septic shock. HRV was analyzed by several methods and the parameters were correlated with pathophysiological processes of sepsis. In 16 anesthetized, mechanically ventilated, and instrumented domestic pigs of either gender, sepsis was induced by fecal peritonitis. Experimental subjects were screened up to the refractory shock development or death. ECG was continuously recorded throughout the experiment, afterwards RR intervals were detected and HRV parameters computed automatically using custom made measurement and analysis MATLAB routines. In all septic animals, progressive hyperdynamic septic shock developed. The statistical measures of HRV, geometrical measures of HRV and Poincaré plot analysis revealed a pronounced reduction of HRV that developed quickly upon the onset of sepsis and was maintained throughout the experiment. The frequency domain analysis demonstrated a decrease in the high frequency component and increase in the low frequency component together with an increase of the low/high frequency component ratio. The reduction of HRV parameters preceded sepsis-associated hemodynamic changes including heart rate increase or shock progression. In a clinically relevant porcine model of peritonitis-induced progressive septic shock, reduction of HRV parameters heralded sepsis development. HRV reduction was associated with a pronounced parasympathetic inhibition and a shift of sympathovagal balance. Early reduction of HRV may serve as a non-invasive and sensitive marker of systemic inflammatory syndrome, thereby widening the therapeutic window for early interventions.

## Introduction

A major cause of health concern that claims a large number of lives worldwide every year are the infectious diseases and related sepsis. Annually, there are 750,000 diagnosed sepsis cases in the USA alone (Angus and van der Poll, 2013) and up to 19 million cases worldwide (estimate by Adhikari et al., 2010). In three most recent prospective studies the mortality of severe sepsis ranged between 18 and 30%, but was much higher in most severe groups (ARISE Investigators et al., 2014; Mouncey et al., 2015). Bacterial peritonitis and consequent abdominal sepsis represent the second most common cause of sepsis-related mortality in the intensive care unit (Sartelli et al., 2014). Mortality rates for secondary peritonitis with severe sepsis or septic shock reach approximately 30% (Sartelli, 2010). Major epidemiological studies have found that due to its progressively increasing incidence (Martin et al., 2003) sepsis is a fundamental medical problem of the new millennium with wide-reaching socio-economic consequences and an enormous burden on the health care system (Tiru et al., 2015). There is thus a critical need to improve our understanding of the (abdominal) sepsis pathophysiology and to develop innovative strategies for early diagnosis and efficacious therapies of this deadly disease.

It was demonstrated by a number of clinical trials that the proper therapy of sepsis should start as soon as possible: early hemodynamic optimization as well as early antibiotic therapy significantly reduced the mortality due to septic shock (Rivers et al., 2001; Kumar et al., 2006). Although the precondition of such therapy is obviously an early and sensitive diagnosis of sepsis, our current diagnostic options are rather limited. Beside the classical sepsis scoring criteria that are based on clinical and inflammatory symptoms together with hemodynamic variables, infection and organ dysfunction, the field of biomarkers holds some promise. The interpretation of biomarker levels, however, is complicated by huge interindividual variability and by dynamics of sepsis progression with a transition from the systemic inflammatory response syndrome to the compensatory anti-inflammatory response syndrome (Tschaikowsky et al., 2002; Osuchowski et al., 2007). Although some molecules are clearly of interest (e.g., presepsin, Wu et al., 2015), for most biomarkers much more detailed understanding of their interactions and functional roles is needed (Kojic et al., 2015).

From other potential indicators of sepsis progression, the HRV receives an increasing attention (Seely and Christou, 2000). The HRV reflects autonomic regulation of the heart and is affected by a number of acute and chronic pathologic conditions including the systemic infection (Gang and Malik, 2002). Depression of HRV in sepsis was reported in both adult (Godin et al., 1996; Korach et al., 2001; Barnaby et al., 2002) and neonate patients (Griffin et al., 2005; Bohanon et al., 2015) and it may have a prognostic value (Chen and Kuo, 2007; Ahmad et al., 2009). Altered autonomic regulation in sepsis may be related to the concept of cholinergic anti-inflammatory pathway (Borovikova et al., 2000; Wang et al., 2004). Despite the accumulating evidence about the potential of HRV to serve as an indicator of sepsis progression and outcome, the relationships of HRV and major pathophysiological processes of sepsis remain unclear. Furthermore, it is not clear what type of HRV analysis provides the most clinically relevant results. Therefore, in this experimental study several domains of HRV were investigated for the first time in a clinically relevant porcine model of peritonitis-induced polymicrobial sepsis that closely mimics human disease. The HRV was analyzed by several methods providing a number of parameters, which were further scrutinized and correlated with pathophysiological processes of sepsis.

## Materials and methods

Animal handling was in accordance with the European Directive for the Protection of Vertebrate Animals Used for Experimental and Other Scientific Purposes (86/609/EU). The experiments were approved by the Committee for Experiments on Animals of the Charles University Faculty of Medicine in Pilsen (protocol No. MSMT-26770/2012-30). All experiments were performed in the animal research laboratory at the Faculty of Medicine in Pilsen. Sixteen domestic pigs of both sexes and of similar weight (39 ± 6 kg) were used for experiments. Sepsis was induced by fecal peritonitis in 11 pigs (six boars, five sows). In five pigs control experiments (analogous procedure but without sepsis induction) were performed to exclude non-specific effects of experimental procedure (surgery, anesthesia, sampling) on the parameters studied.

### Anesthesia and instrumentation

The experimental animals were kept fasting for 18 h prior to the each experiment with unrestrained access to water. Anesthesia was induced with i.m. ketamine (2 mg/kg) and azaperone (2–4 mg/kg) and i.v. propofol 2% (1–2 mg/kg). Orotracheal intubation was performed and mechanical ventilation was initiated (FiO2 0.3, PEEP 6 cm H2O, tidal volume 8 ml/kg, respiratory rate was adjusted to maintain end/tidal pCO2 between 4 and 5 kPa). During the instrumentation, surgical anesthesia was maintained with continuous i.v. propofol (1–4 mg/kg/h) and fentanyl (10–15 μg/kg/h). Muscle paralysis was induced and maintained with i.v. norcuronium (4 mg for induction, 0.2–0.4 mg/kg/h for maintenance). After the instrumentation anesthesia with propofol (1–4 mg/kg/h) and fentanyl (5–10 μg/kg/h) was maintained until the end of the experiment. Infusion of Ringerfundin solution (B. Braun Melsungen AG, Melsungen, Germany) 10 ml/kg/h was administered during surgical procedures and then reduced to 7 ml/kg/h as a maintenance fluid. Normoglycemia (arterial blood glucose level 4.5–7 mmol/l) was maintained throughout the whole experiment using 20% glucose infusion as needed.

All pigs were instrumented using femoral artery catheter enabling continuous blood pressure monitoring and blood sampling. Triple lumen central venous catheter and pulmonary artery catheter were both inserted via exposed jugular veins for hemodynamic monitoring. Two silicone drains were inserted to Morrison and Douglas anatomical spaces via middle laparotomy in order to enable feces inoculation.

### Experimental protocol

After the surgical preparation a 6-h phase of recovery was allowed before baseline measurements. In the sepsis group, peritonitis was induced by inoculating 1 g/kg of autologous feces (collected pre-operatively and suspended in 200 mL 37°C isotonic saline) into the abdominal cavity through the drainage tubes. In addition to continuous crystalloid solution infusion, fluid boluses (10 ml/kg of Ringerfundin) were administered to maintain normovolemia in a goal-directed fashion guided by filling pressures and cardiac output (CO) response. Continuous i.v. norepinephrine was administered if mean arterial pressure (MAP) fell below 65 mmHg and titrated to maintain MAP above 70 mmHg.

### Measurements and calculations

Measurements of hemodynamics included CO, systemic vascular resistance (SVR), filling pressures of both ventricles (CVP, PAOP). The plasma levels of tumor necrosis factor-alpha (TNF-α) and interleukin 6 (IL-6) were determined with immunoassays in arterial blood obtained just before and 12, 24, and 36 h after induction of sepsis. Electrocardiogram (lead II) was recorded using Biopac System (Biopac Systems Inc., Santa Barbara, CA, USA). The sampling rate was 1000 Hz. In the control group (without the induction of sepsis) two 5-min recordings were performed: after the 6-h recovery phase and 24 h later. In the septic group the ECG started 30 min before the induction of sepsis and continued for next 44 h or until the animal died. These long-term ECG recordings were divided into 1-h sections in which the HRV analysis was executed separately, i.e., all HRV measures were computed every single hour to describe sepsis development throughout the experiment.

### Heart rate variability analysis

All ECG recordings were analyzed offline in MATLAB 2014b (MathWorks Inc., Natick, MA, USA, 2014). R peaks were detected using derivative-threshold algorithm. RR intervals were checked and ectopic beats were manually excluded. On RR interval series several computational methods were applied to obtain statistical, geometrical, Poincaré plot, and frequency domain parameters (Task Force of the European Society of Cardiology and the North American Society of Pacing and Electrophysiology, 1996). Statistical parameters included standard deviation of all RR intervals (SDNN), standard deviation of differences between adjacent RR intervals (SDSD), percentage of pairs of successive intervals that differ by more than 50 ms (pNN50) and root mean square of successive RR interval differences (RMSSD). Geometrical parameters were based on the histogram of RR intervals measured on a discrete scale with bins of 1/128 s. HRV triangular index (HRV TI) was obtained as total number of all RR intervals divided by the height of the histogram and TINN as baseline width of the minimum square difference triangular interpolation of the highest peak of the histogram. Poincaré plot [dependence of the *n*^*th*^ RR interval on the (*n*–1)^*th*^ RR interval] was fitted by an ellipse with semiaxes of length equal to SD1, resp. SD2. SD1 was determined as standard deviation of points perpendicular to the identity line and SD2 as standard deviation of points along the identity line. Area of the Poincaré plot ellipse was computed as *S* = π·*SD*1· SD2. To quantify cardiac autonomic regulation during sepsis progression frequency domain analysis was performed. Lomb-Scargle periodogram was used to estimate the power spectral density. Power in the low frequency range (LF, absolute or normalized units; 0.04–0.15 Hz) and high frequency range (HF, absolute or normalized units; 0.15–0.40 Hz) were determined.

### Statistical analysis

Results are presented as means ± SD. After testing for normality of distribution (Shapiro-Wilk test), statistical comparisons were made with One-way ANOVA with repeated measures followed by *post-hoc* Bonferroni and Dunnett tests. The analysis was performed using software package STATISTICA Cz, version 8 (StatSoft CR s.r.o., Prague,CZ, 2007), and Origin 8.5 (OriginLab Corp., Northampton, MA,USA, 2011). Differences at *p* < 0.05 were considered significant. Analysis of time courses of the heart rate and HRV was obtained using software OriginPro 8.5.

## Results

**Figure 1:**
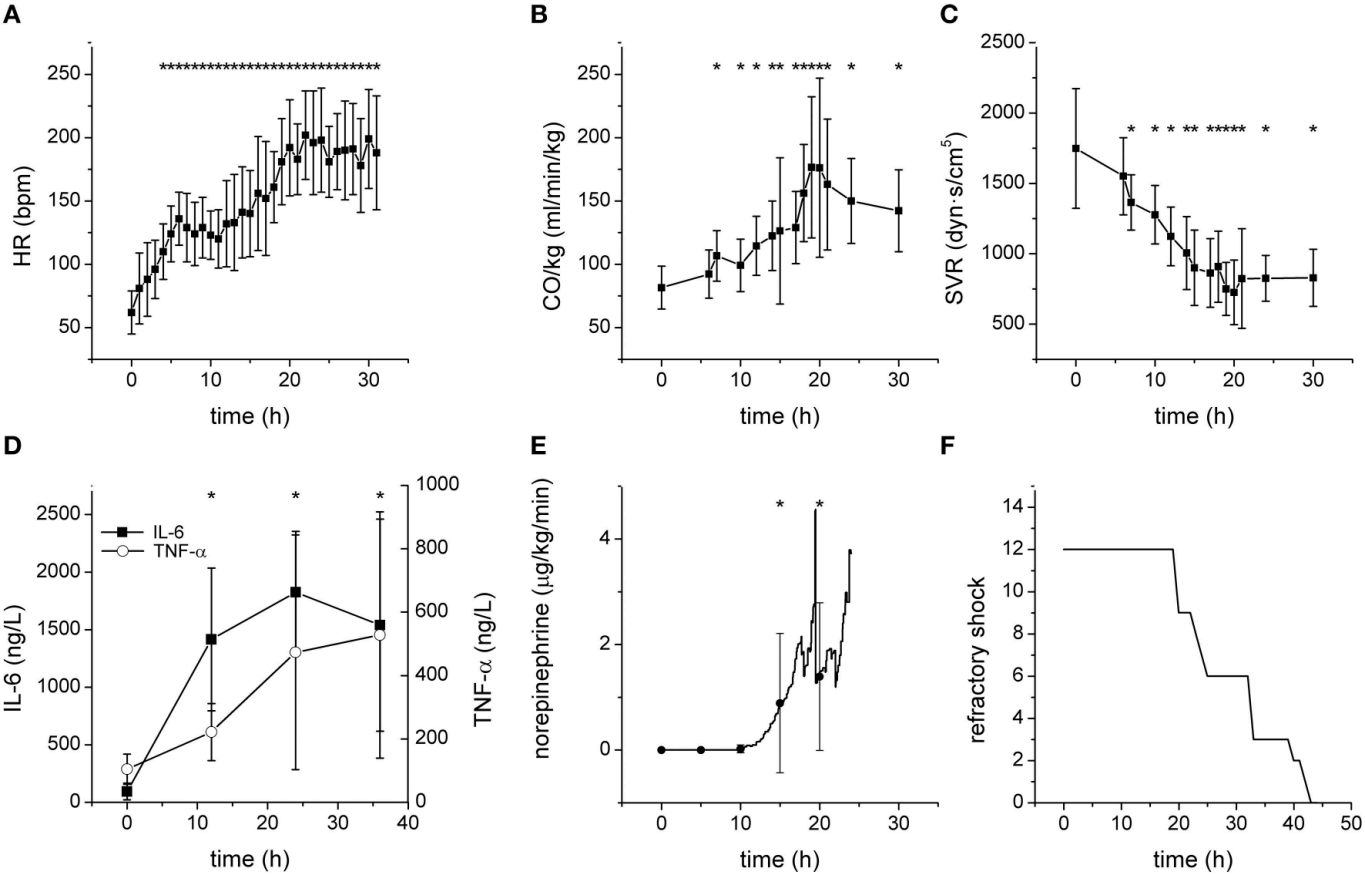
**Characteristics of the porcine model of severe sepsis/septic shock**. ^*^Significantly different from baseline (*p* < 0.05). **(A)** Heart rate (bpm) during sepsis progression. **(B)** Cardiac output (normalized to the body weight) during sepsis progression. **(C)** Systemic vascular resistance during sepsis progression. **(D)** Plasma levels of IL-6 and TNF-α during sepsis progression. **(E)** Therapeutic doses of norepinephrine during sepsis progression. **(F)** Development of refractory shock/death of septic pigs.

All septic animals developed hyperdynamic circulation with increased heart rate, CO (Figures 1A,B), and reduced SVR (Figure 1C). The plasma levels of inflammatory mediators IL-6 and TNF-α were significantly increased (Figure 1D). All septic pigs needed norepinephrine to maintain MAP above 70 mmHg (Figure 1E). Refractory shock leading to the animal's death developed in septic animals between 19 and 43 h from the induction of fecal peritonitis (Figure 1F).

**Figure 2:**
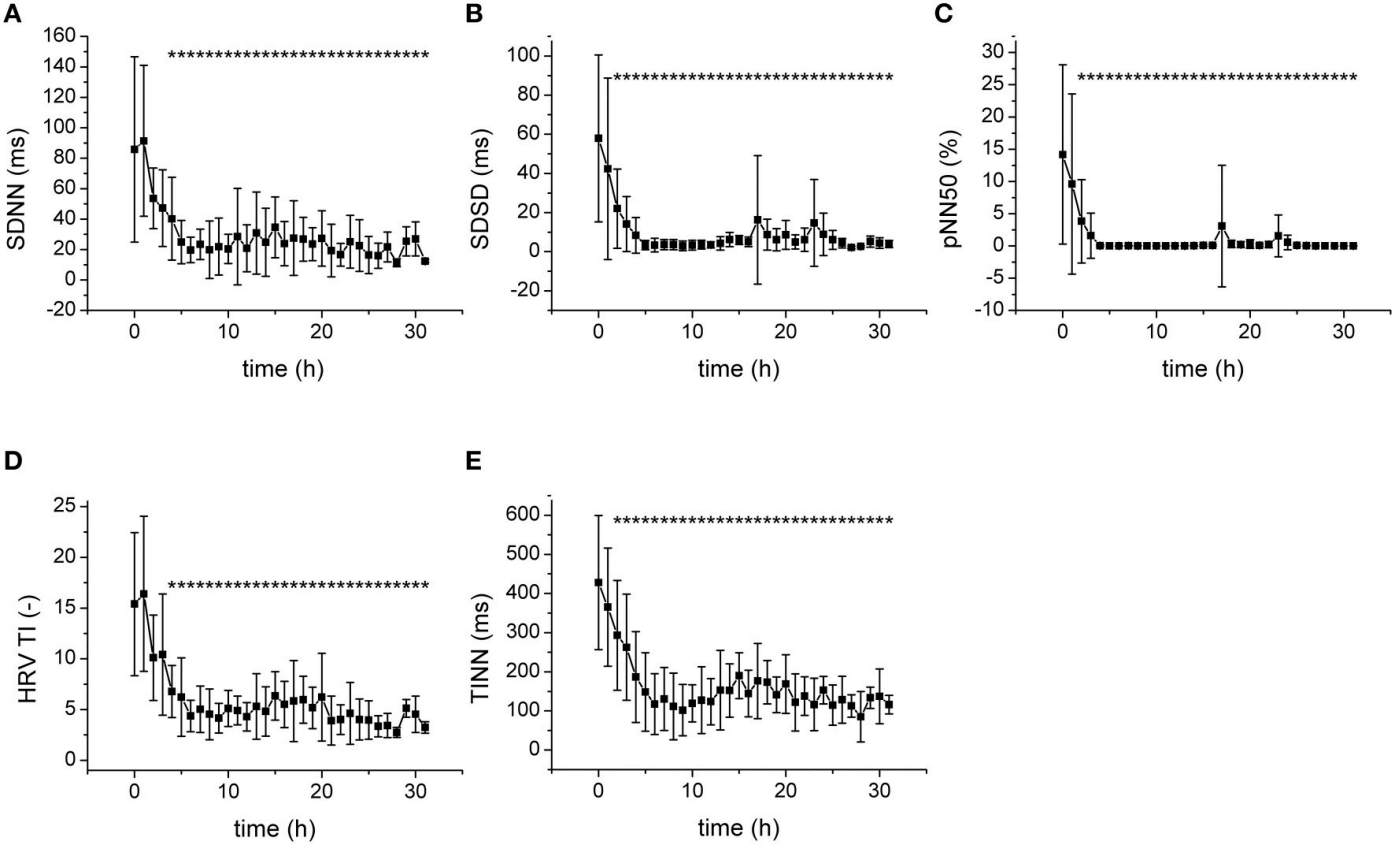
**Statistical and geometrical parameters of HRV**. ^*^Significantly different from baseline (*p* < 0.05). **(A)** SDNN, standard deviation of RR intervals, during sepsis progression. **(B)** SDSD, standard deviation of differences between adjacent RR intervals, during sepsis progression. **(C)** pNN50, percentage of pairs of successive intervals that differ by more than 50 ms, during sepsis progression. **(D)** HRV TI, total number of all intervals divided by the height of the histogram, during sepsis progression. **(E)** TINN, baseline width of the minimum square difference triangular interpolation, during sepsis progression.

**Figure 3:**
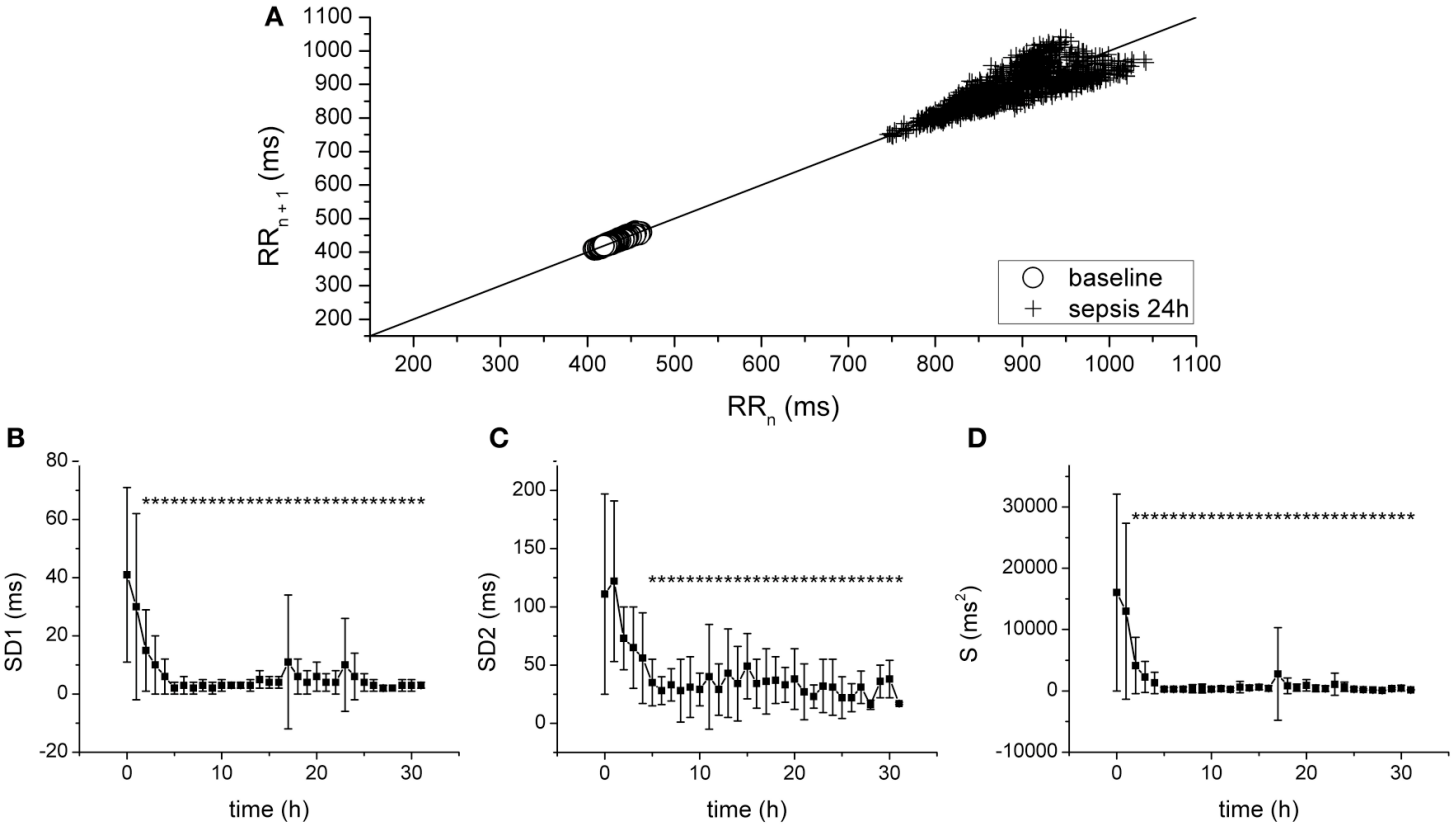
**Analysis of HRV with Poincaré plots. (A)** Poincaré plot of RR intervals at baseline (crosses) and after 24 h of sepsis development (circles). **(B)** SD1, SD of points perpendicular to the identity line, during sepsis progression. ^*^Significantly different from baseline (*p* < 0.05). **(C)** SD2, SD of points along the identity line, during sepsis progression. ^*^Significantly different from baseline (*p* < 0.05). **(D)** S, area of the fitted ellipse, during sepsis progression. ^*^Significantly different from baseline (*p* < 0.05).

Progression of fecal peritonitis/sepsis was associated with a marked reduction of the HRV as documented by several types of analysis. The reduction was revealed by time domain statistical parameters SDNN (Figure 2A), SDSD (Figure 2B) pNN50 (Figure 2C), and RMSSD (not shown) as well as by geometrical parameters obtained by the analysis of RR interval histogram HRV TI (Figure 2D) and TINN (Figure 2E). The beat-to-beat variability of RR intervals was analyzed using Poincaré plots (Figure 3). The reduction of variability in sepsis was again documented by very narrow clustering of the data points measured 24 h after induction of peritonitis (corresponds to severe sepsis/septic shock) compared to the large baseline cluster (Figure 3A). Clustering of the data points was quantified by parameters SD1 (Figure 3B), SD2 (Figure 3C), and S (Figure 3D) and all showed a marked reduction with sepsis progression.

**Figure 4:**
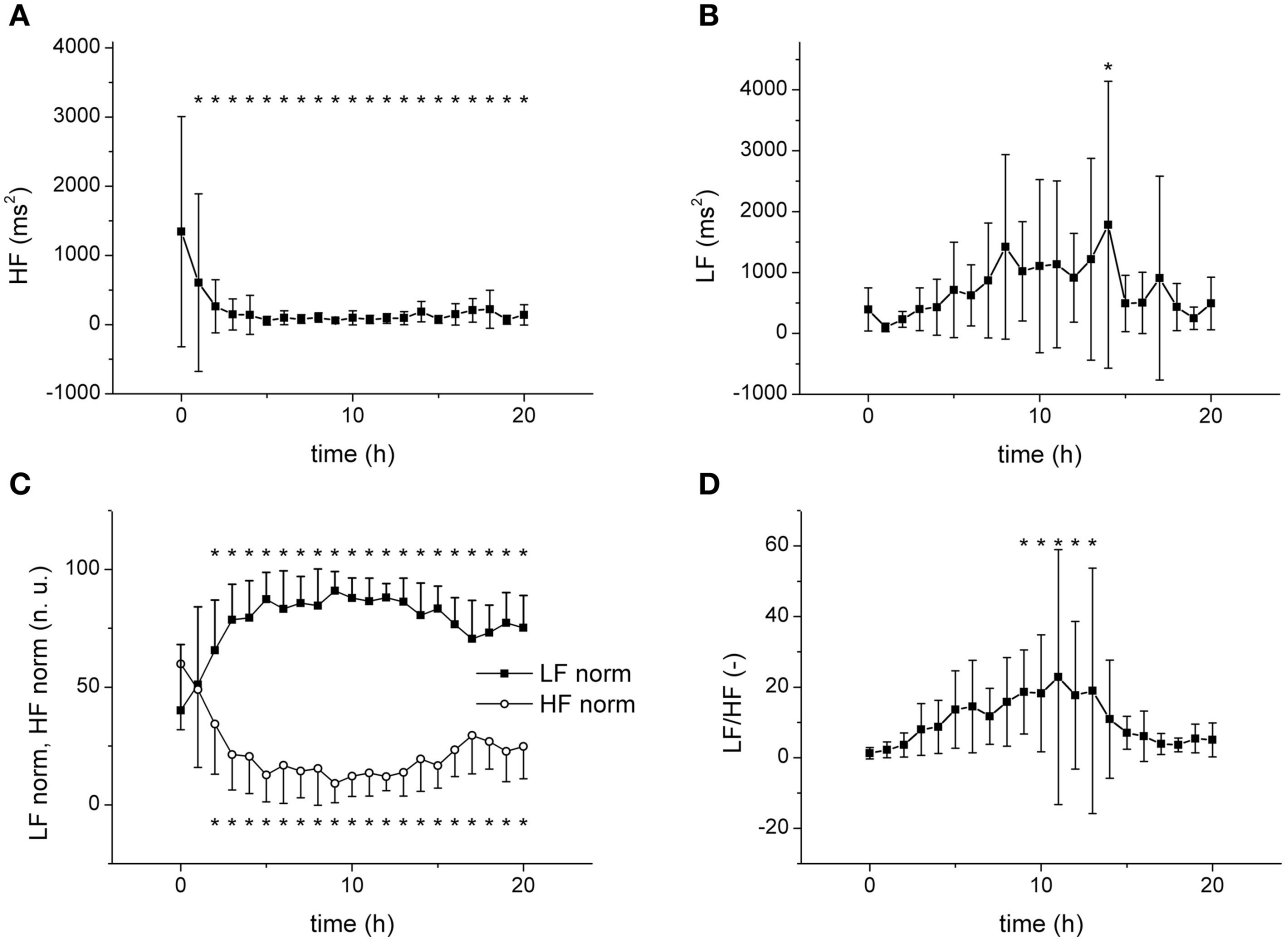
**Frequency domain analysis of HRV**. ^*^Significantly different from baseline (*p* < 0.05). **(A)** Power of high frequency band (HF) during sepsis progression. **(B)** Power of low frequency band (LF) during sepsis progression. **(C)** Normalized HF and LF components during sepsis progression. **(D)** Low-frequency/high-frequency band ratio (LF/HF) during sepsis progression.

The frequency domain analysis revealed a substantial and fast (time constant of 1.77 ±1.35 h) reduction of the power of the high-frequency band (HF) by sepsis (Figure 4A), whereas the power of the low-frequency band (LF) was elevated by sepsis only transiently (at 14 h, Figure 4B). When the HF and LF power components were normalized (Figure 4C), a clear increase of LF and a decrease of HF with similar dynamics were found. The low-frequency/high-frequency band ratio (LF/HF) showed a transient increase between 9 and 13 h (Figure 4D).

In control experiments (without the induction of sepsis) the variability parameters (both time and frequency domains) were not altered and remained similar throughout the entire 24-h-lasting experiment (e.g., SDNN of 4.746 ± 2.033 ms at baseline vs. 6.872 ± 3.991 ms at 24 h, SD1 of 4.26 ± 2.391 ms at baseline vs. 3.332 ± 2.982 ms at 24 h, LF of 45.546 ± 46.342 ms^2^ at baseline vs. 44.964 ± 65.464 ms^2^ at 24 h).

**Figure 5:**
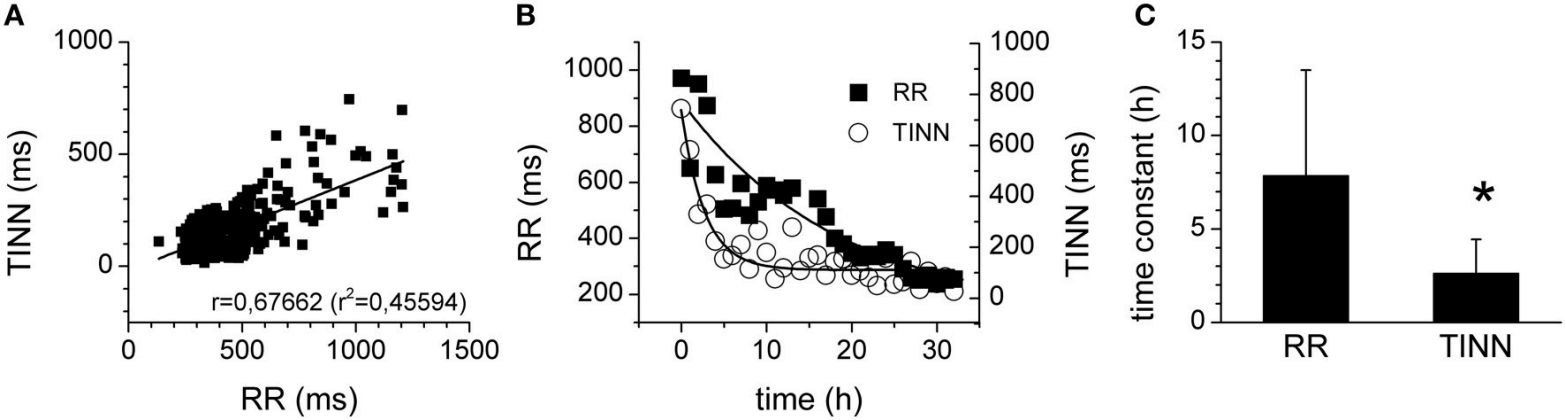
**Relationship of HRV and heart rate. (A)** Correlation of TINN (geometrical parameter of HRV) and of RR interval. The straight line represents the linear fit of the data points. **(B)** Time courses of RR intervals (full squares) and of TINN parameters (open circles). The curves represent the exponential fits of the data points. **(C)** Time constants of the RR interval and TINN parameter kinetics. ^*^Significantly different (*p* < 0.05).

Since the HRV is significantly associated with average heart rate and it is not easy to determine which of these two plays the principal role in the prognostic value of the HRV (Sacha et al., 2013), the relationship between heart rate and HRV was investigated in more detail. Indeed, a significant correlation was found for RR interval and for a HRV parameter, TINN (*r* = 0.67662, *R*^2^= 0.45594, *p* = 0.02581; Figure 5A). Analysis of the time courses of the heart rate and of HRV (parameter TINN) showed, however, that the kinetics of the HRV reduction were much faster than those of the RR interval Figures 5B,C). Similar results were obtained for all other HRV parameters measured (e.g., time constant of 2.17 ±1.23 h for SDNN, time constant of 2.79 ±1.09 h for HRV TI, time constant of 2.07 ±1.36 h for SD1; *p* > 0.05, when the time constants of all parameters compared).

**Figure 6:**
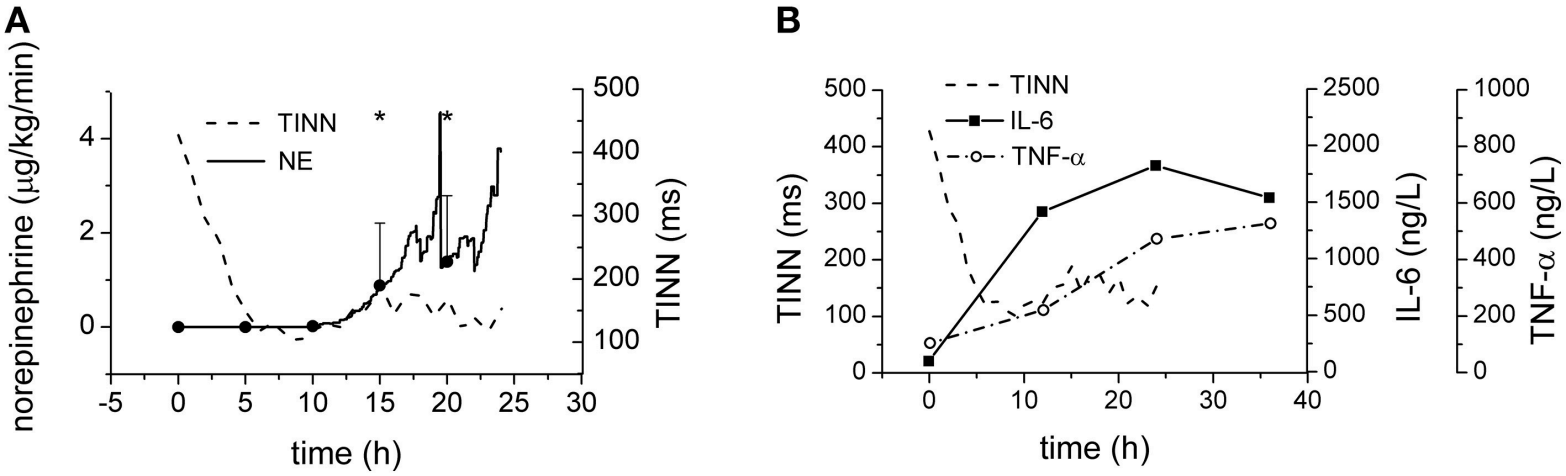
**Norepinephrine and cytokines during sepsis progression. (A)** Therapeutic doses of norepinephrine during sepsis progression. Full line, average doses of norepinephrine. Dashed line, time course of TINN parameter of HRV. **(B)** Plasma levels of IL-6 (full line) and of TNF-α (dash-and-dotted line) during sepsis progression. Dashed line, time course of TINN parameter of HRV. ^*^Significantly different from baseline (*p* < 0.05).

Therapeutic application of norepinephrine could significantly affect the cardiovascular system in general and perhaps contribute to the HRV reduction (Tulppo et al., 2001). The Figure 6A shows, however, that the reduction of the HRV is completed several hours before the administration of norepinephrine was started indicating that the HRV reduction is an intrinsic phenomenon independent of the therapeutic norepinephrine administration. Similarly, the elevations of plasma levels of inflammatory mediators IL-6 and TNF-α (Figure 6B) seem slower than kinetics of HRV parameters.

**Figure 7:**
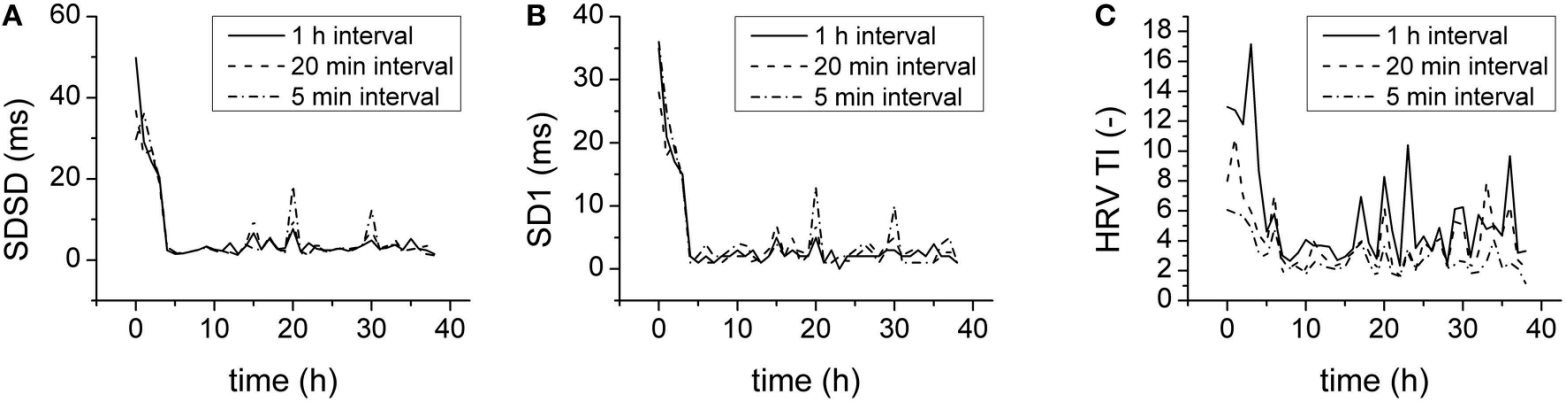
**Duration of analysis and HRV parameters. (A)** SDSD for 1 h (full line), 20 min (dashed line), and 5 min (dash-and-dotted line) analysis intervals. **(B)** SD1 for 1 h (full line), 20 min (dashed line), and 5 min (dash-and-dotted line) analysis intervals. **(C)** HRV TI for 1 h (full line), 20 min (dashed line), and 5 min (dash-and-dotted line) analysis intervals.

The HRV parameters above were obtained by analysis of 1 h-lasting intervals. To find a compromise between the computational expense of analysis and the physiological relevance, parameters were also computed for shorter intervals (20 min, 5 min) and compared. For statistical and Poincaré plot parameters even 5-min-lasting intervals provided results similar to those obtained with 1-h-lasting intervals (Figures 7A,B). The geometrical parameters, however, were reduced by shortening analysis intervals (Figure 7C).

**Figure 8:**
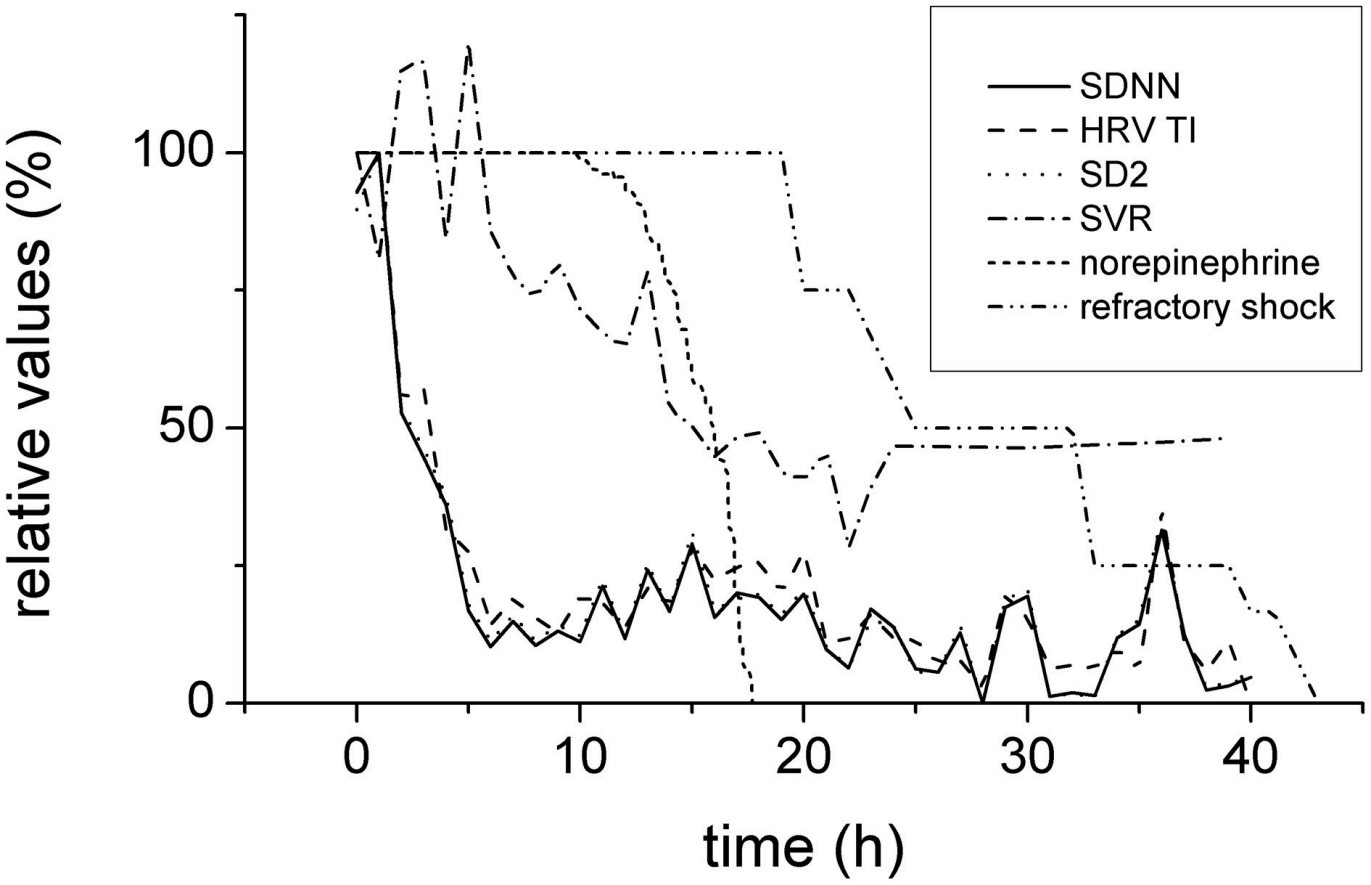
**Time relationships of HRV, hemodynamics, and refractory shock development**. Expressed in relative values (100% corresponds to baseline). Depression of HRV parameters (SDNN, full line, HRV TI, dashed line, and SD2, dotted line) preceded the hemodynamic instability (systemic vascular resistance, dash-and-dotted line; reverse norepinephrine dose calculated as a difference of the respective dose and baseline 100%, short-dashed line) and refractory shock/death (double-dot-and-dashed line).

In order to enable comparison of the predictive value of several processes, their percentual changes of baseline values were plotted together (Figure 8). Clearly, the reduction of the heart variability parameters was the earliest (completed approximately 5 h from the induction of peritonitis) and preceded the hemodynamic instability (manifested by reduced SVR and therapeutic administration of norepinephrine) by several hours.

## Discussion

In a clinically relevant large animal model of progressive septic shock an early and marked reduction of HRV parameters preceded the onset of overt hemodynamic alterations thus heralding sepsis progression and providing a significant therapeutic window for early therapeutic interventions. The reduction of HRV probably results from autonomic nervous dysbalance with reduced parasympathetic cardiac modulation and a shift of the sympathovagal balance toward sympathetic cardiac modulation.

In general, the results of this study confirmed the potential of the HRV to serve as an early and sensitive indicator of sepsis progression as suggested by other studies (Godin et al., 1996; Korach et al., 2001; Barnaby et al., 2002; Griffin et al., 2005; Chen and Kuo, 2007; Ahmad et al., 2009; Bohanon et al., 2015). The HRV received in the last decade an intensive attention especially in association with the late-onset neonatal sepsis. Early diagnosis of neonatal sepsis is difficult since the clinical signs are non-specific and non-uniform and blood cultures have a substantial false-negative rate (Griffin et al., 2007). In the infants, in which the sepsis develops, typically the HRV is reduced and transient decelerations occur (Griffin and Moorman, 2001). Based on these abnormal heart rate (variability) characteristics, the heart rate characteristics index was developed and validated in neonatal intensive care units (Griffin et al., 2003, 2004, 2005, 2007). The HRV analysis in neonatal sepsis was repeatedly shown to add independent information to clinical signs scores and to allow for earlier diagnosis of the sepsis (Griffin et al., 2007; Bohanon et al., 2015). In adult patients, HRV was shown to be altered by sepsis (Korach et al., 2001; Barnaby et al., 2002) and several studies suggested a significant prognostic value of HRV parameters in sepsis (Ellenby et al., 2001; Chen and Kuo, 2007; Chen et al., 2008; Ahmad et al., 2009).

The frequency domain analysis revealed a decrease of the HF band and an increase in the LF band (especially when normalized) resulting in a transient increase of the LF/HF ratio suggesting a substantial reduction of parasympathetic cardiac modulation and a shift of the sympathovagal balance toward sympathetic cardiac modulation. In general, there is a considerable discrepancy regarding the autonomic regulation of the heart (rate variability) in sepsis. Several studies documented impaired sympathovagal balance with a low LF/HF ratio in septic patients (Korach et al., 2001; Barnaby et al., 2002). In contrast, Ellenby et al. (2001) demonstrated by observing serial changes in HRV of seven children with septic shock an increase over time in the LF component and the LF/HF ratio, whereas the HF component was decreased. Similarly, a transient increase in the LF/HF ratio was shown in patients with sepsis and septic shock (Papaioannou et al., 2009). In experimental animal studies, an increase in the LF component and the LF/HF ratio was shown in endotoxemic rats (Huang et al., 2010). In endotoxemic rabbits, both LF and HF components initially after endotoxin administration trended upward and then decreased as hypotension developed (Goldstein et al., 1995). Although these discrepancies may be related to a number of factors (e.g., species, severity of disease, age, type of shock), the time factor is probably of importance and dynamic changes of the sympathovagal balance during sepsis progression could contribute to the controversial experimental results.

Our results indicate that the sympathovagal balance in the porcine model of severe sepsis/septic shock shifts toward sympathetic activation. This is an interesting finding with regard to the concept of the cholinergic anti-inflammatory pathway, according to which the nervous system via an inflammatory reflex of the vagus nerve can inhibit cytokine release and thereby prevent sepsis progression (Tracey, 2007). Reduction of the HF component together with an increase in the LF/HF ratio suggest that the vagal cholinergic system including the cholinergic anti-inflammatory pathway is in this porcine model insufficiently activated and/or inhibited, which could contribute to the fast sepsis development toward refractory septic shock.

All time domain and non-linear (Poincaré plot) parameters decreased with similar kinetics (full reduction reached in approximately 5 h, *p* > 0.05, when time constants of all parameters compared). The frequency domain analysis revealed that the dominant reduced band was the parasympathetic HF component, which showed similar (fast) dynamics of the reduction (*p* > 0.05, when time constants compared). On the other hand, the LF band tended to increase, but with much slower kinetics and only transiently (only significant after 14 h). Seemingly discrepant fast increase of the normalized LF component must be attributed to the reduction of HF component, which is involved in the calculation of normalized LF. The data indicate that the parasympathetic inhibition is the dominant mechanism of HRV reduction in porcine sepsis. The sympathetic contribution is probably minor and, with regard to its slow dynamics, perhaps secondary to hemodynamic changes. The transient nature of the LF/HF ratio elevation may reflect a later suppression of cardiac sympathetic nervous modulation by therapeutically administered norepinephrine.

Beside the autonomic nervous regulation of the heart, recent studies suggest that intrinsic cardiac mechanisms (i.e., cardiac pacemaking) should also be taken into account (Papaioannou et al., 2013). The cardiac pacemaking in the sinoatrial node is the result of a complex interaction of multiple ionic currents including the pacemaker I_*f*_ current. This current was reported to be depressed by endotoxin (Zorn-Pauly et al., 2007; Klöckner et al., 2014), thus possibly contributing to the reduction of HRV in sepsis. In conditions of prolonged elevated catecholamine levels with subsequent peripheral cardiac desensitization as documented in sepsis (Tang and Liu, 1996; Annane et al., 1999) one could speculate that the relative contribution of the intrinsic cardiac mechanisms to the HRV would increase.

Possible contribution of exogenously administered norepinephrine to the reduction of the HRV and to the shift of the sympathovagal balance was excluded by comparing the time courses of these events (Figures 6, 8). The changes of the HRV developed well before norepinephrine administration and therefore other mechanisms must be involved. In the later stages of the septic shock, however, some contribution of high doses of therapeutic norepinephrine cannot be excluded. Similarly, also the dynamics of major inflammatory cytokines TNF-α and IL-6 seem slower than the dynamics of HRV parameters. The plasma levels of TNF-α and IL-6 continued rising after 12 h from the induction of peritonitis whereas the reduction of HRV parameters was completed in less than 10 h from the induction of peritonitis. It should be realized, however, that from technical reasons the blood sampling interval (12 h) was quite long and some early fast changes could be missed. This is especially the case of IL-6, rise of which was faster than that of TNF-α. Since in other studies IL-6 was reported to peak 2–4 h after the endotoxin challenge (Fong et al., 1989; Durosier et al., 2015) and to correlate well with fetal HRV measures in a fetal sheep model of lipopolysaccharide-induced sepsis (Durosier et al., 2015), its possible contribution should be analyzed in more detail.

HRV is significantly correlated with average heart rate on both mathematical and physiological bases (Sacha et al., 2013). Since during sepsis both heart rate and HRV are altered, the question arises whether or to what extent the changes of the heart rate contribute to the changes of the HRV. In this study a correlation of the HRV and the heart rate was found, however the kinetics of the HRV were faster than those of the heart rate, thus arguing against a major role of the average heart rate in the alteration of the HRV in sepsis. Similar dissociation between the changes in the heart rate and the HRV with faster kinetics of the HRV was found in mice with polymicrobial sepsis due to cecal ligation and puncture (Hoover et al., 2015).

When the HRV was analyzed in intervals of various duration (1 h, 20 min, 5 min), similar results were obtained for statistical and Poincaré plot parameters. The geometrical parameters (obtained by the analysis of histogram of RR intervals), however, were reduced by shortening the analysis interval below 20 min. Therefore, when considering a proper compromise between the computational expense of analysis and the physiological relevance, 5 min intervals seem sufficient for statistical and Poincaré plot parameters but not for the histogram geometrical parameters, which require longer data segments for analysis (Task Force of the European Society of Cardiology and the North American Society of Pacing and Electrophysiology, 1996). When intervals of proper duration were analyzed, all assessed parameters were able to demonstrate the reduction of HRV and none of them was superior indicating that the reduction of HRV in sepsis is indeed a robust signal that can be visualized by various types of HRV analysis.

## Study limitations

The results of the study do not allow elucidating causal relationships between the HRV and the progression of disease. The reduction of HRV is, according to our data, dominantly related to diminished parasympathetic cardiac modulation, which would negatively affect the cholinergic anti-inflammatory pathway. Therefore, the cholinergic anti-inflammatory pathway may represent the link between the HRV and systemic inflammation. Despite considerable evidence accumulated in support of this concept, a number of issues remain unresolved and will require further investigation (Olofsson et al., 2012).

Direct effects of inflammatory mediators on cardiac pacemaker cells of the sinoatrial node and/or remodeling of pacemaker cells may be involved in the phenomenon (Papaioannou et al., 2013). Strong autonomic dysbalance evidenced by the frequency domain analysis, however, argues against larger contributions of these mechanisms. Screening multiple inflammatory mediators and consequent detailed analysis of ionic currents during slow diastolic depolarization in sinoatrial node cells is beyond the scope of this study.

For the analysis of HRV the classical time and frequency domain analyses together with non-linear Poincaré plot analysis were used. Other advanced techniques for analysis of non-stationary data like multiscale analysis (Gao et al., 2013) were not employed in this study although their clinical potential was demonstrated (Hu et al., 2009, 2010; Gao et al., 2012). These sophisticated techniques could be helpful especially in the early phases of the disease when the cardiovascular disturbances are still subtle. Multiscale analysis of the early HRV changes with the aim of further widening of the therapeutic window for early interventions is the task for future studies.

Domestic pigs of both sexes were used for the experiments. Although no sex differences in HRV (both at baseline and during sepsis) were detected, with regard to low numbers of animals they cannot be completely excluded. Similarly, effects of anesthesia on HRV cannot be ruled out. We assume, however, that although the absolute values may differ between conscious and anesthetized animals, the pattern of HRV reduction in sepsis should be similar.

## Conclusions

In a clinically relevant porcine model of peritonitis-induced progressive septic shock, reduction of HRV developed well before the onset of overt clinical sepsis. This fast and pronounced HRV reduction was associated with a pronounced parasympathetic inhibition and consequent shift of cardiac sympathovagal balance toward sympathetic cardiac modulation. Monitoring HRV could provide a promising tool for early diagnosis of infection-induced systemic inflammatory and hemodynamic alterations and widening the therapeutic window for early treatment.

## Author contributions

DJ performed electrocardiographic experiments, developed the Matlab routines, analyzed HRV, performed the statistical analysis and drafted the manuscript. LV participated in the *in vivo* hemodynamic studies and performed the statistical analysis. JC participated in the *in vivo* hemodynamic studies, in the coordination of the study and performed the statistical analysis. JB participated in the *in vivo* hemodynamic studies, performed the statistical analysis and helped to draft the manuscript. JS, BF, and LN participated in HRV analysis and interpretation including statistical analysis. MM and MS conceived of and designed the study, participated in the coordination of the study and data analysis and drafted the manuscript. All authors contributed to revising the work and approved the final manuscript.

### Conflict of interest statement

The authors declare that the research was conducted in the absence of any commercial or financial relationships that could be construed as a potential conflict of interest.
